# Insights into the draft genome sequence of bioactives-producing *Bacillus thuringiensis* DNG9 isolated from Algerian soil-oil slough

**DOI:** 10.1186/s40793-018-0331-1

**Published:** 2018-10-11

**Authors:** Mohamed Seghir Daas, Albert Remus R. Rosana, Jeella Z. Acedo, Malika Douzane, Farida Nateche, Salima Kebbouche-Gana, John C. Vederas

**Affiliations:** 1 Valcore Laboratory, Department of Biology, University M’Hamed Bougara of Boumerdes, 35000 Boumerdes, Algeria; 2 Food Technology Research Division, Institut National de la Recherche Agronomique d’Algérie, 16200, El Harrach, Algiers, Algeria; 3 Department of Chemistry, University of Alberta, Edmonton, AB T6G 2G2 Canada; 4 Microbiology Group, Laboratory of Cellular and Molecular Biology, Faculty of Biological Sciences, University of Science and Technology–Houari Boumediene, 16111, Bab Ezzouar, Algiers, Algeria

**Keywords:** *Bacillus thuringiensis*, Genome sequencing, Bioinformatics, Secondary metabolites, Bacteriocin, Zwittermycin a

## Abstract

*Bacillus thuringiensis* is widely used as a bioinsecticide due to its ability to form parasporal crystals containing proteinaceous toxins. It is a member of the *Bacillus cereus* sensu *lato*, a group with low genetic diversity but produces several promising antimicrobial compounds. *B. thuringiensis* DNG9, isolated from an oil-contaminated slough in Algeria, has strong antibacterial, antifungal and biosurfactant properties. Here, we report the 6.06 Mbp draft genome sequence of *B. thuringiensis* DNG9. The genome encodes several gene inventories for the biosynthesis of bioactive compounds such as zwittermycin A, petrobactin, insecticidal toxins, polyhydroxyalkanoates and multiple bacteriocins. We expect the genome information of strain DNG9 will provide another model system to study pathogenicity against insect pests, plant diseases, and antimicrobial compound mining and comparative phylogenesis among the *Bacillus cereus* sensu *lato* group.

**Electronic supplementary material:**

The online version of this article (10.1186/s40793-018-0331-1) contains supplementary material, which is available to authorized users.

## Introduction

*Bacillus thuringiensis* is a rod-shaped, Gram-positive bacterium that has been isolated from a variety of ecological niches including soil, aquatic environments, and dead insects, among many others [1]*.* *B. thuringiensis* is known for its utility as a bioinsecticide due to its ability to produce parasporal crystals that contain protein toxins (e.g. Cry proteins, also called δ-endotoxins) during its sporulation and stationary growth phase [2]. These protein toxins have also been successfully introduced to genetically modified crops, as exemplified in Bt corn, rendering these crops resistant to specific insect pests [3]. The protein toxins have been shown to be safe to plants, beneficial insects, and mammals due to the absence of specific receptors that are normally only found in the target organisms [4, 5]. The potential of *B. thuringiensis* to serve as an alternative to chemical insecticides has driven the discovery of new *B. thuringiensis* strains that may lead to the identification of novel protein toxins with potential use in pest management [1, 6]. Aside from the insecticidal properties of *B. thuringiensis**,* it has also been reported to exhibit antibacterial, antifungal, antibiofilm and emulsifying activities [7, 8]. In general, the *Bacillus* species are known to be rich sources of antimicrobial compounds [9–12]. For *B. thuringiensis**,* its antibacterial effects can be attributed to a wide range of compounds including bacteriocins and lipopeptides [13]*.* On the other hand, its antifungal activity has been attributed to the production of compounds such as zwittermycin, chitinase, and lipopeptides [7]. In this study, the whole genome sequence of *B. thuringiensis* DNG9 that was isolated from an oil-contaminated slough in Baraki-Algiers, Algeria was determined. This strain was chosen for sequencing due to its strong antimicrobial and emulsifying properties. It was the aim of this work to obtain a better understanding of the observed bioactivities based on the genes encoded in its genome.

## Organism information

### Classification and features

**Fig. 1:**
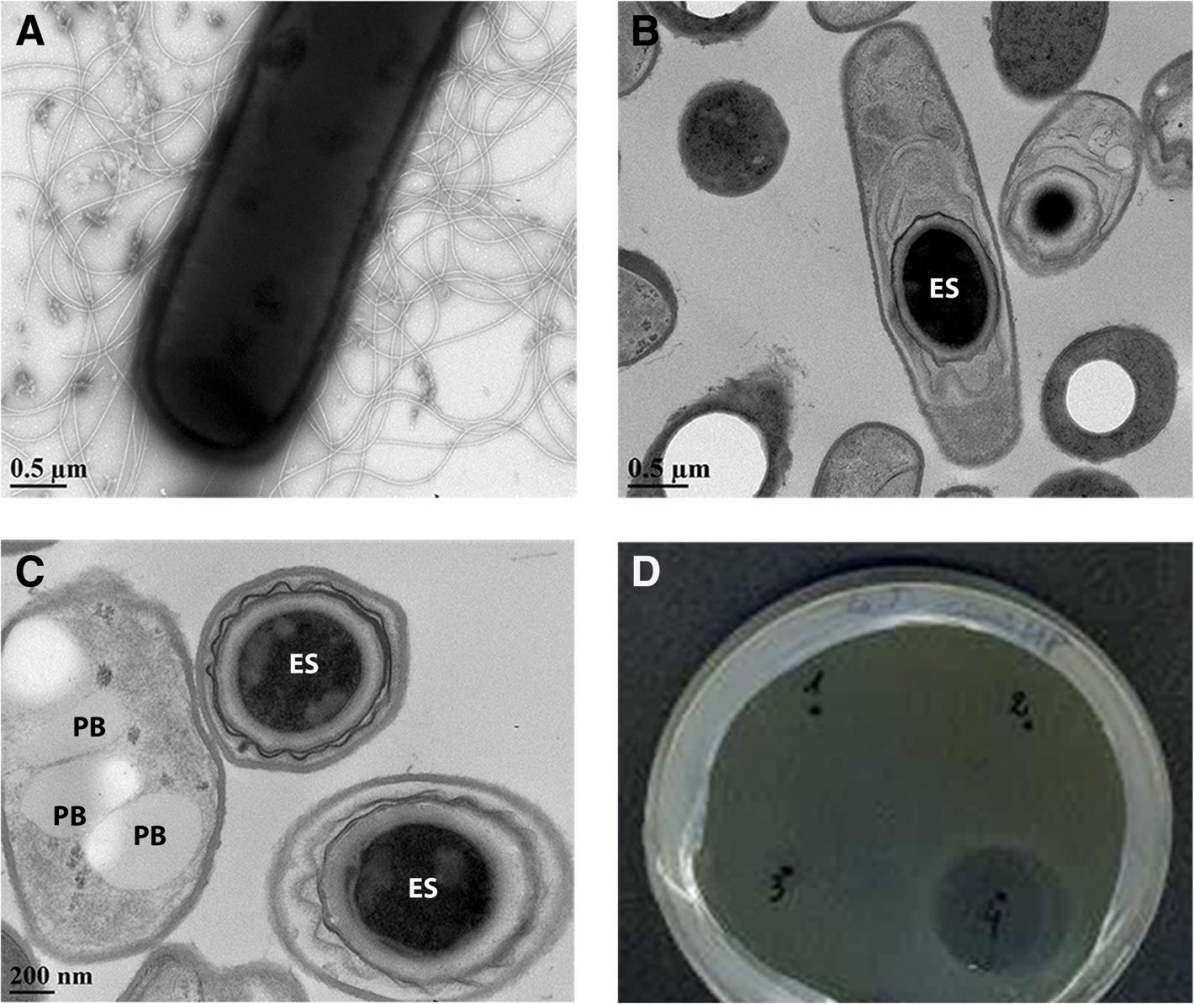
General characteristics of *Bacillus thuringiensis* DNG9. Transmission electron micrograph (TEM) of DNG9 showing **a** flagellated cell, **b** subcentral endospore, ES, and **c** parasporal bodies, PB. **d** Spot-on-lawn assay showing the activity of DNG9 supernatant (labelled as 4) against indicator strain *Lactococcus lactis subsp. cremoris* HP

*Bacillus thuringiensis* DNG9 was isolated from an oil-contaminated soil slough in Baraki-Algiers, Algeria. The samples were serially diluted in water, heat-shocked at 80 °C for 30 min, spread onto Luria Bertani (LB) agar and incubated at 35 °C for 24 h. Strain DNG9, like the majority of other reported *B. thuringiensis* strains, are Gram-positive, aerobic to facultative anaerobic bacterium [14]. The cells are rod-shaped, flagellated (Fig. 1a) and endospore-forming (Fig. 1b, c). The bacterium has a growth temperature range from 10 to 48 °C with an optimal growth at 28–35 °C [15] and pH 4.9–8.0 with an optimal pH of 7.0 [16, 17]. It produces parasporal bodies during the stationary phase of its growth cycle (Fig. 1c), which is consistent with the three *cry* genes predicted from its genome. Two homologs of *cry41* and one homolog of *cry6* genes were predicted from the genome of DNG9 using the BtToxin Scanner server [18]. The key features of DNG9 are summarized in Table 1.

**Table 1:** Classification and general features of *Bacillus thuringiensis* strain DNG9 according to the MIGS recommendation [19]

MIGS ID	Property	Term	Evidence code^a^
	Classification	Domain Bacteria	TAS [53]
		Phylum *Firmicutes*	TAS [16]
		Class *Bacilli*	TAS [54, 55]
		Order *Bacillales*	TAS [42, 56]
		Family *Bacillaceae*	TAS [42, 57]
		Genus *Bacillus*	TAS [41, 42]
		Species *Bacillus thuringiensis*	TAS [42, 58]
		Strain DNG9	
	Gram stain	Positive	IDA
	Cell shape	Rod	IDA
	Motility	Motile	IDA
	Sporulation	Spore (Subcentral)	IDA
	Temperature range	10 °C – 48 °C	TAS [15]
	Optimum temperature	28 °C – 35 °C	TAS [15]
	pH range; Optimum	4.9–8.0; 7.0	TAS [16, 17]
	Carbon source	Glucose	NAS
MIGS-6	Habitat	Soil	NAS
MIGS-6.3	Salinity	Salt tolerant	TAS [59]
MIGS-22	Oxygen requirement	Aerobic,	IDA
MIGS-15	Biotic relationship	Free-living	IDA
MIGS-14	Pathogenicity	Insect pathogen	TAS [60]
MIGS-4	Geographic location	Algeria	NAS
MIGS-5	Sample collection	February 13, 2013	NAS
MIGS-4.1	Latitude	36° 40′ 9″ N	NAS
MIGS-4.2	Longitude	3° 5′ 43″ E	NAS
MIGS-4.4	Altitude	22 m	NAS

^a^Evidence codes: *IDA* Inferred from Direct Assay, *TAS* Traceable Author Statement (i.e., a direct report exists in the literature), *NAS* Non-traceable Author Statement (i.e., not directly observed for the living, isolated sample, but based on a generally accepted property for the species, or anecdotal evidence). These evidence codes are from the Gene Ontology project [61]

**Fig. 2:**
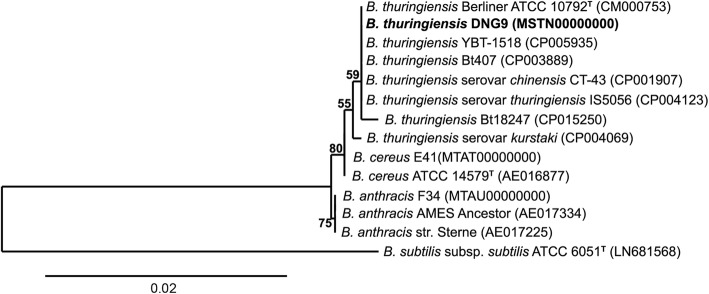
Maximum likelihood phylogeny of Bacillus thuringiensis DNG9 16S rRNA gene isolated from Algerian soil-oil slough. Nucleic acid sequences were aligned using Geneious and the tree compiled using RaxML. Numbers above the branches refer to bootstrap values. The tree was rooted using *Bacillus subtilis subsp. subtilis* ATCC 6051^T^. Type strains are indicated with ^T^. All strains represent sequenced genomes. Scale bar indicates 2 nucleotide substitution for each 10 nucleotide sequences. Accession numbers of publicly available sequences are given in brackets

Thirteen *Bacillus* strains and DNG9 were chosen for phylogenetic analysis. The chosen species represent the members of *B. cereus* sensu *lato* supergroup [19]. This includes the type strains *B. thuringiensis* Berliner ATCC 10792^T^, *B. cereus* ATCC 14579^T^ and *B. anthracis* AMES Ancestor. The 16S rRNA gene sequence from the type strain *B. subtilis* subsp. *subtilis* ATCC 6051^T^ [20] was selected as an outgroup. The maximum likelihood method was used to construct the phylogenetic tree shown in Fig. 2. The phylogenetic tree supports the placement of strain DNG9 within the *B. thuringiensis* group together with the type strain *B. thuringiensis* Berliner ATCC 10792^T^.

## Genome sequencing information

### Genome project history

The project information and associated MIGS (Minimum Information about a Genome Sequence) 2.0 compliance [21] are summarized in Table 2. This bacterium was selected for sequencing as it was determined to be one of the most promising strains for discovery of compounds with strong antibacterial (Fig. 1d), antifungal and biosurfactant abilities (Additional file 1: Figure S1). The availability of the draft genome of DNG9 may contribute to the evolution and comparative genomics studies of the *B. cereus* sensu *lato* group. Furthermore, future investigations on its genome-encoded bioactive metabolites may be pursued. This work provided a standard draft genome and the assembled contigs have been deposited in public repositories. The PGAP- and JGI-IM- annotated genomes were deposited to the DDBJ/ENA/GenBank databases under accession numbers MSTN00000000 and Ga0180945, respectively.

**Table 2:** Project information

MIGS ID	Property	Term
MIGS 31	Finishing quality	Draft genome
MIGS-28	Libraries used	Illumina paired-end
MIGS 29	Sequencing platforms	Illumina MiSeq100
MIGS 31.2	Fold coverage	317×
MIGS 30	Assemblers	CLC Genomic Workbench 7.5.2
MIGS 32	Gene calling method	GeneMarkS, Prodigal
	Locus Tag	BVF97
	Genbank ID	MSTN00000000
	GenBank Date of Release	9-Mar-17
	GOLD ID	Ga0180945
	BIOPROJECT	PRJNA359364
MIGS 13	Source Material Identifier	DNG9
	Project relevance	Agricultural, Biotechnological

### Growth conditions and genomic DNA preparation

Genomic DNA was isolated from a combined 16-h grown single colony isolate and a two mL 16-h grown liquid culture (150 rpm) from LB agar and LB broth, respectively. Total nucleic acid was extracted using the method described previously [22]. Briefly, cells were harvested at 500×g for 2 min and resuspended in 100 μl 1× TE buffer (100 mM Tris-HCl, 50 mM EDTA, pH 8.0). Cell slurry was sequentially treated with 20 mg/ml lysozyme (37 °C, 30 min), 2 mg/ml proteinase K (56 °C, 30 min) and 0.5 mg/ml RNase A (37 °C, 30 min). The sphaeroplast suspension was lysed with 500 μl cell breakage buffer (0.4% sodium dodecyl sulfate, 0.5% N-lauroyl sarcosine, 0.5% Triton X-100, 50 mM Tris, 100 mM EDTA, pH 8.0), 400 μl phenol and 150 μl glass beads (0.5 mm dia, Sartorius, Germany). The slurry was vortexed for 1 min and rested for 1 min on ice, for a total of 10 cycles, and finally clarified at 13000×g for 5 min at room temperature. The aqueous layer was repeatedly extracted with equal volume of phenol, followed by phenol:chloroform (1:1) and finally with chloroform:isoamyl alcohol (24:1). The DNA was precipitated with 0.1× 3 M sodium acetate pH 5.2 and 2.5× absolute ethanol, washed with 70% ethanol and resuspended in 10 mM Tris buffer, pH 8.0. Quantity and quality were assessed using Qubit 2.0 fluorometry (Qiagen) and agarose gel electrophoresis, respectively.

### Genome sequencing and assembly

The genome of *Bacillus thuringiensis* DNG9 was sequenced at The Applied Genomic Core, Department of Biochemistry, University of Alberta using Illumina paired-end sequencing platform and Nextera XT DNA library kit (Illumina, USA). Whole genome sequencing was performed in duplicates using the MiSeq Reagent kit v2. Sequencing of 250 bp paired-end modules gathered 3.69 M reads, which provided an average coverage of 317× resulting in 38 contigs. De novo assembly of the 6,057,430 bp paired-end sequences was created using CLC Genomics Worksbench v 7.5.2. (CLC bio, Aarhus, Denmark).

### Genome annotation

Gene prediction was performed using four automated genome annotation pipelines: (1) the NCBI Prokaryotic Genome Annotation Pipeline (PGAP) [23] using GeneMarkS+ and best-placed reference protein set; (2) the Joint Genome Institute – Integrated Microbial Genomes and Microbiomes (JGI-IMG/M) pipeline [24] utilizing Prodigal gene caller [25]; (3) the Rapid Annotation using Subsystem Technology (RAST) v2.0 server [26]; and (4) the Bacterial Annotation System (BASys) server [27]. CRISPR repeats were predicted by using CRISPRfinder [28]. The draft genome of DNG9 was aligned with the type strain *B. thuringiensis* Berliner ATCC 10792^T^ closed genome to generate a single scaffold using Contiguator v2 [29] and Multi-Draft based Scaffolder (MEDUSA) [30]. A chromosome map was generated from the single scaffold using BASys automated pipeline [27] and viewed using CGViewer [31].

Species was established using genome-wide Average Nucleotide Identity (gANI) metric and alignment fraction (AF) calculated within the JGI-IMG/M server using the Microbial Species Identifier (MiSI) calculator [32]. Strain was established using the Genome-to-Genome Distance Calculator (GGDC) 2.1 server employing digital DNA:DNA hybridization (dDDH) and DNA G + C content [33].

## Genome properties

**Fig. 3:**
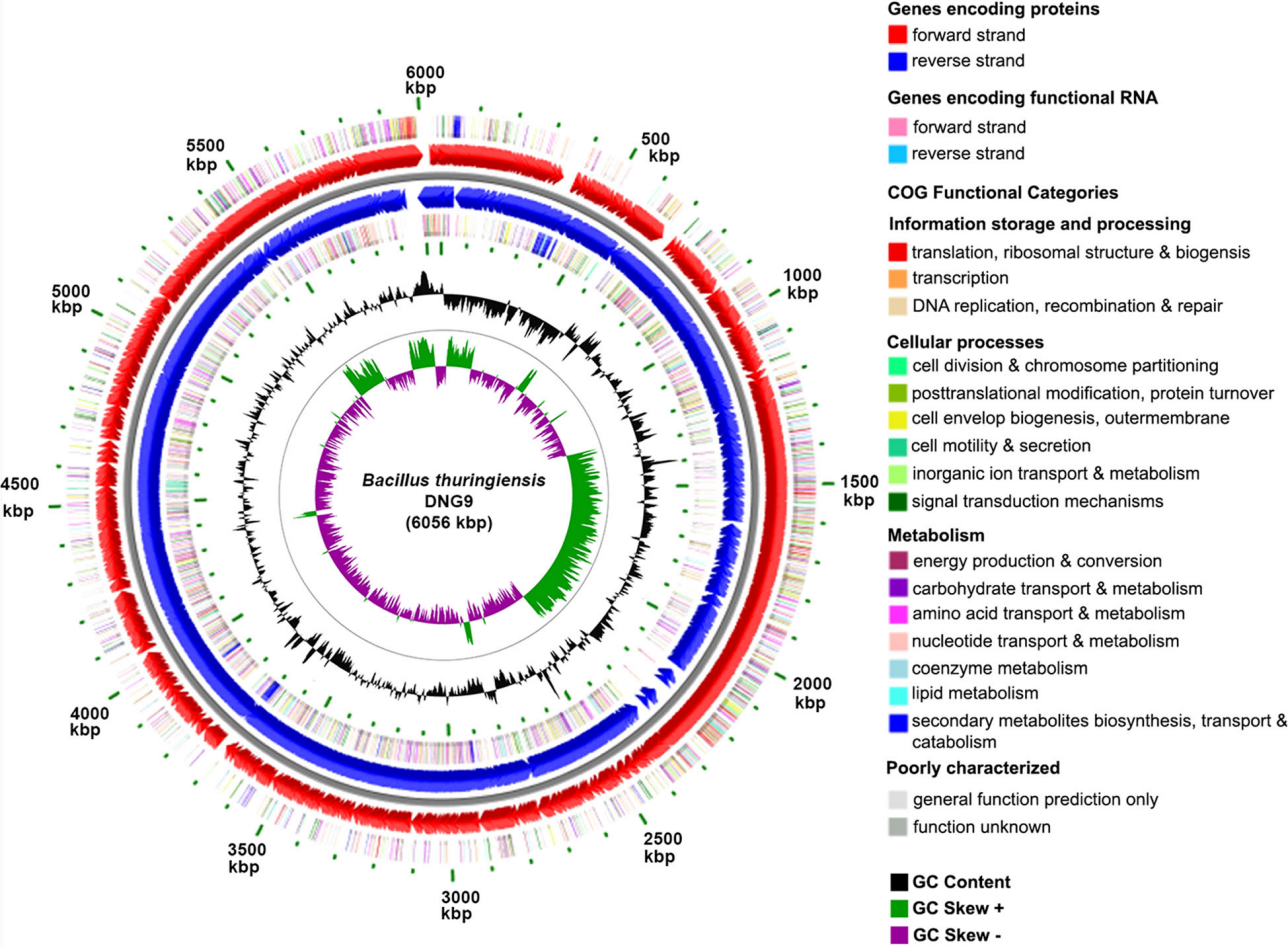
Circular representation of the draft genome of DNG9 representing relevant genome features. The draft genome was aligned into one scaffold using *B. thuringiensis* Berliner ATCC 10792^T^ genome. The outer most circle shows COG functional categories of coding regions in the clockwise direction. The lines in each concentric circle represent the position of the indicated feature; the color legend is shown to the right of the map. The second circle shows predicted coding regions transcribed on the forward (clockwise) DNA strand. The third circle shows predicted coding regions transcribed on the reverse (counterclockwise) DNA strand. The fourth circle shows COG functional categories of coding regions in the counterclockwise direction. The fifth and sixth circles show the percent GC content of the genome and the percent GC deviation (skewness) by strand, respectively

The draft genome of DNG9 is 6,057,430 bp with 34.9% GC content, similar to the genomes of other *Bacillus thuringiensis* strains [34–36], and contained 38 scaffolds with N_50_ of 347,259 bp. A total of 135 RNA genes and 284 pseudogenes were annotated by IMG/M and PGAP, respectively (Table 3). Annotation using the DOE-JGI IMG/M pipeline revealed 6109 total coding sequences of which 4463 have functional predictions. Conversely, RAST annotation pipeline predicted 6055 coding sequences; NCBI-PGAP revealed 6213 coding genes; and lastly, BASys annotated 6102 coding sequences. The 4463 coding sequences predicted in IMG/M pipeline were placed in 25 general clusters of orthologous (COG) functional gene catalogs. The distribution of these protein-coding genes based on COG function is listed in Table 4. The 6.06 Mbp draft genome map of DNG9, as aligned against the type strain *B. thuringiensis* Berliner ATCC 10792, is presented in Fig. 3.

**Table 3:** Genome statistics

Attribute	Value	% of Total
Genome size (bp)	6,057,430	100.00
DNA coding (bp)	5,053,197	83.42
DNA G + C (bp)	2,107,907	34.80
DNA scaffolds	38	100.00
Total genes	6109	100.00
Protein coding genes	5974	97.79
RNA genes	135	2.21
Pseudo genes	284	4.65
Genes in internal clusters	2024	33.13
Genes with function prediction	4463	73.06
Genes assigned to COGs	3633	59.47
Genes with Pfam domains	4883	79.93
Genes with signal peptides	284	4.65
Genes with transmembrane helices	1741	28.50
CRISPR repeats	4	0.07

**Table 4:** Number of genes associated with general COG functional categories

Code	Value	%age	Description
J	262	6.38	Translation, ribosomal structure and biogenesis
A	0	0	RNA processing and modification
K	388	9.44	Transcription
L	135	3.29	Replication, recombination and repair
B	1	0.02	Chromatin structure and dynamics
D	60	1.46	Cell cycle control, Cell division, chromosome partitioning
V	124	3.02	Defense mechanisms
T	213	5.19	Signal transduction mechanisms
M	236	5.74	Cell wall/membrane biogenesis
N	55	1.34	Cell motility
U	36	0.88	Intracellular trafficking and secretion
O	160	3.89	Posttranslational modification, protein turnover, chaperones
C	210	5.11	Energy production and conversion
G	250	6.09	Carbohydrate transport and metabolism
E	400	9.74	Amino acid transport and metabolism
F	130	3.16	Nucleotide transport and metabolism
H	228	5.55	Coenzyme transport and metabolism
I	146	3.55	Lipid transport and metabolism
P	233	5.67	Inorganic ion transport and metabolism
Q	109	2.65	Secondary metabolites biosynthesis, transport and catabolism
R	3.96	9.64	General function prediction only
S	301	7.33	Function unknown
–	2476	40.53	Not in COGs

The total is based on the total number of protein coding genes in the genome

## Insights from the genome sequence

*B. thuringiensis* DNG9 was found to be flagellated, sporulating with a subcentral endospore and producing the insecticidal parasporal bodies (Fig. 1a, b, c). These phenotypes are supported by gene inventories found in the genome of DNG9 (Fig. 3). The RAST annotation has allocated these genes into 490 subsystems, the most abundant of which are genes that are associated with amino acid and derivatives metabolism (15.5%), followed by carbohydrate (11.7%), and protein metabolism (7.6%).

**Fig. 4:**
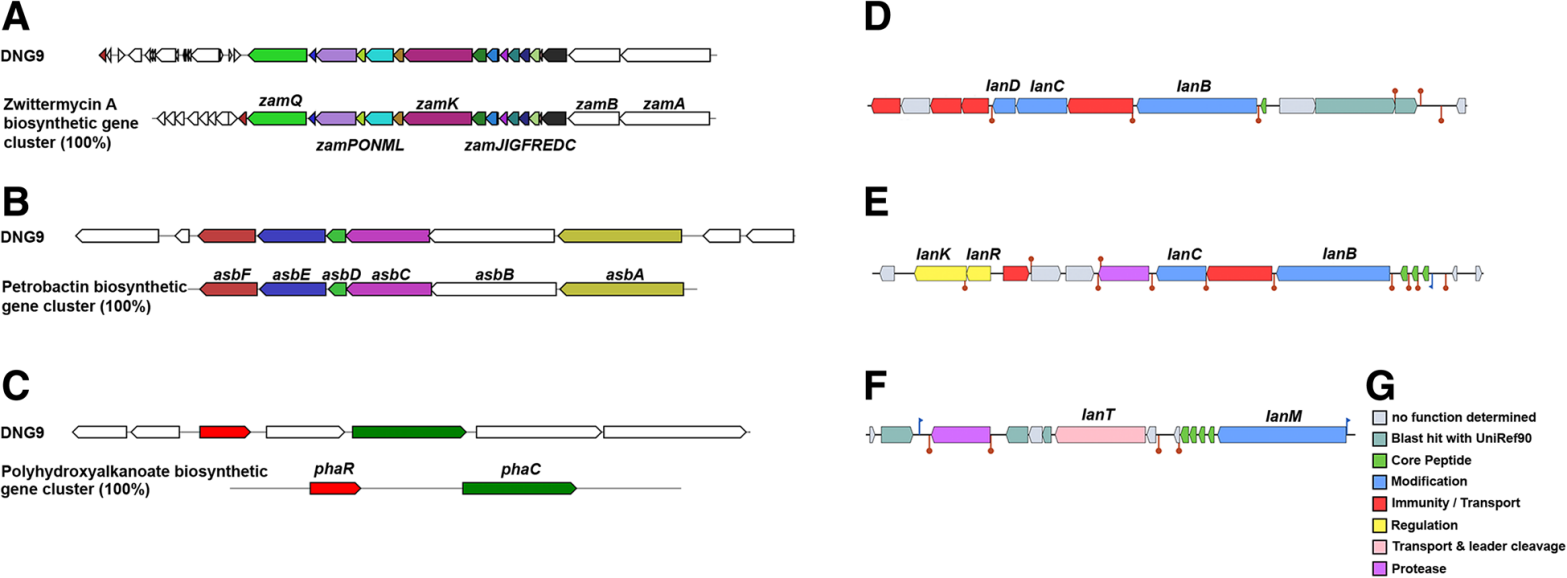
Secondary metabolite biosynthetic gene cluster organization in DNG9. Gene clusters for zwittermycin A (**a**), petrobactin (**b**), and polyhydroxyalkanoate (**c**) biosynthesis as predicted by antiSMASH 4.0. The DNG9 biosynthetic gene cluster is color coded with respect to its homology (%) to the known biosynthetic gene cluster. Gene cluster for three lanthipeptide class I (**d**), lanthipeptide class I (**e**) and lanthipeptide class II (**f**) biosynthesis as predicted by BAGEL 4.0. Color legend for Fig. 4d, e, f is presented in G

DNG9 was found to be most active against *Lactococcus lactis subsp. cremoris* HP (Fig. 1d) [37, 38], and was also active against *Carnobacterium divergens* LV13 [39], *Salmonella. enterica* Typhimurium ATCC 23564 [40], and *Micrococcus* sp. ATCC 700405 [41] but not against *Escherichia coli* JM109 [42, 43], *Pseudomonas aeruginosa* ATCC 14217 [42, 44], and *Enterococcus faecalis* 710C [45]. Conversely, DNG9 was also found to be active against the fungus *Galactomyces* *geotrichum* MUCL 28959 but not *Aspergillus niger* ATCC 9142 and *Candida albicans* ATCC 10231. The antiSMASH 4.0 server predicted that DNG9 genome carries the gene clusters responsible for the production of several secondary metabolites including antibiotics, siderophores, and biopolymers. The genome was found to encode gene clusters with complete homology to the biosynthetic gene clusters of the antifungal compound, zwittermycin A (Fig. 4a), the iron-siderophore, petrobactin (Fig. 4b), and the bioplastic precursor, polyhydroxyalkanoates (PHAs) (Fig. 4c). The aminopolyol compound zwittermycin A was previously shown to suppress fungal-oomycete diseases in plants [46, 47], suggesting that the antifungal activity of DNG9 could be attributed to this secondary metabolite. The presence of siderophores, like petrobactin and bacillibactin, in the genome of DNG9 suggests its iron acquisition abilities. These gene clusters are not exclusive in *B. thuringiensis* but are also found in the genomes of other members of the *Bacillus cereus* sensu *lato* group [48–50]. Both antiSMASH 4.0 and BAGEL 4.0 servers also predicted a number of novel bacteriocins, mainly belonging to the class referred to as lanthipeptides (Fig. 4d, e, f). Lastly, Bt_toxin scanner revealed that *cry* genes encoding the insecticidal protein associated with *B. thuringiensis* is present in DNG9 genome*,* two homologs of *cry41* and one homolog of *cry6* genes. The wide biological target range of DNG9, including its antibacterial, antifungal and insecticidal properties, could be attributed to these bioactive compounds.

**Fig. 5:**
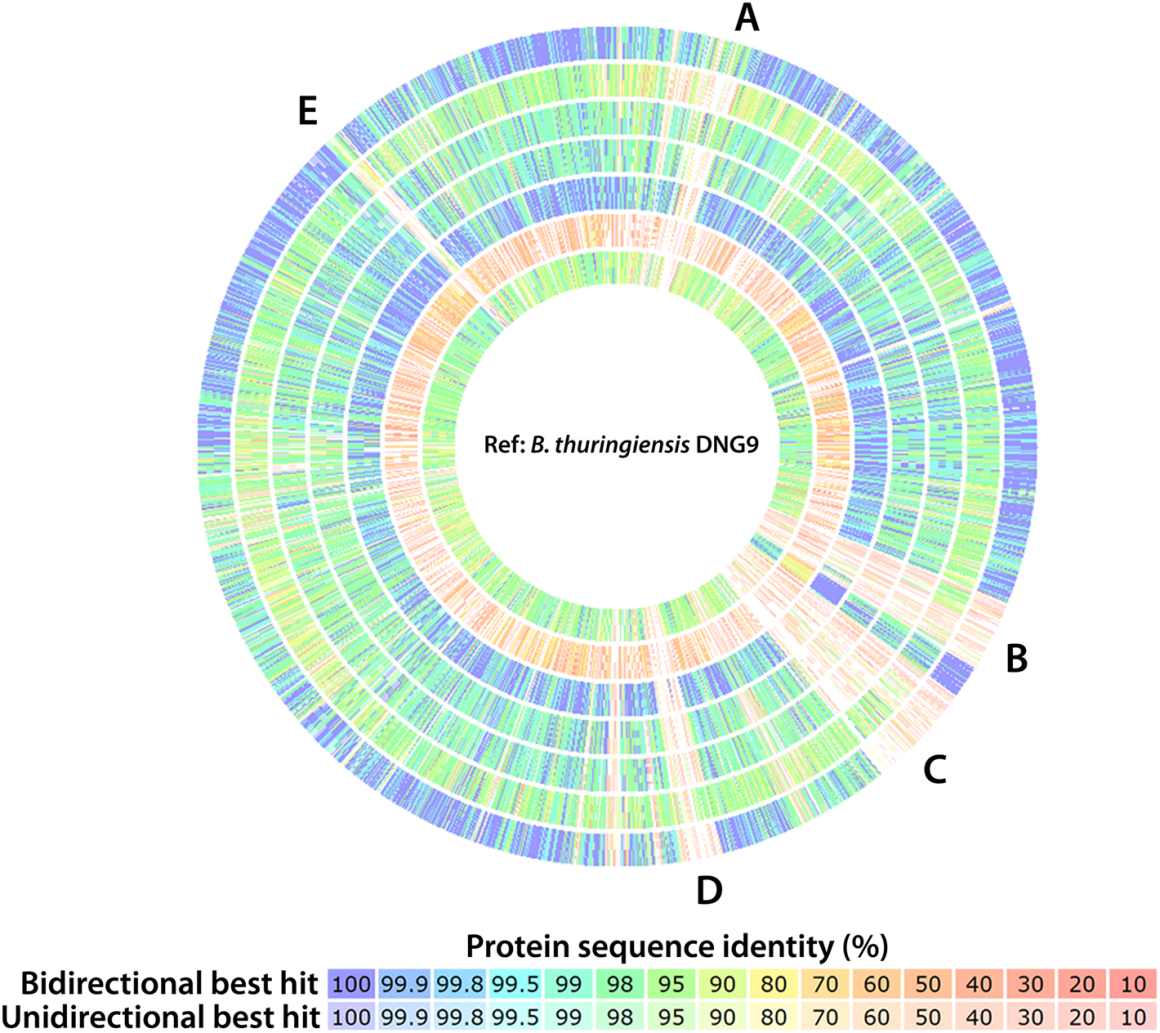
Genomic comparison of DNG9 to other Bacillus sp. genomes conducted using RAST. Each track represents pair-wise BLAST comparison between the open reading frames in query genome against those in *Bacillus thuringiensis* DNG9 (Ref. = reference), with percentage of similarity represented with different colors shown in the legend. Regions marked in the genomic map correspond to gene number presented in Additional File 3: Table S2 (**a** = 250–313, **b** = 1882–2051, **c** = 2127–2374, **d** = 2785–2880, **e** = 5318–5365). Query genomes used in this analysis (outer ring to inner ring): *B. thuringiensis* Berliner ATCC 10792^T^, *B. anthracis* F34, *B. cereus* ATCC 14579, *B. cereus* E41, *B. thuringiensis* YBT-1518, *B. subtilis subsp. subtilis* ATCC 6051^T^ and *B. anthracis* AMES Ancestor

The genome of DNG9 is highly similar to those of *B. thuringiensis* Berliner ATCC 10792^T^, *B. thuringiensis* YBT-1518, and *B. thuringiensis* Bt407 based on average nucleotide identity (> 99%) and digital DNA:DNA hybridization (> 95%) (Additional file 2: Table S1), shared gene content (Fig. 5) and phylogenetic analyses of the 16S rRNA gene (Fig. 2). The functional comparison of DNG9 genome composition with closely related *Bacillus* species (i.e. *B. thuringiensis**,* *B. cereus* and *B. anthracis*) [19] is presented in Fig. 5. *Bacillus subtilis subsp. subtilis* ATCC 6051^T^ was used as an outgroup in the map. Comparison of the genomes of DNG9 and seven closely related *Bacillus* species by uni- and bidirectional best BlastP implemented in RAST, cross-validated with IMG annotations and viewed in IslandViewer 4 server [51], revealed strain-specific genes that encode hypothetical proteins, which are grouped into genomic islands. (Fig. 5, Additional file 3: Table S2). These ORFs in DNG9 include a high proportion of mobile genetic elements, phage-like proteins, transposases and hypothetical proteins in five distinct genomic islands including an intact prophage in region A which is further supported by Phaster server [52] analysis.

## Conclusions

In conclusion, here we report a 6.06 Mbp draft genome of *Bacillus thuringiensis* DNG9, isolated from an oil-contaminated soil-slough in Baraki-Algeirs, Algeria. The final de novo assembly is based on 306.5 Mb of Illumina data, which provided an average coverage of 317×. The assembled genome contains 6120 coding sequences (average of 4 annotation pipelines), of which the most abundant are genes that are associated with amino acid (15.5%), followed by carbohydrate (11.7%), and protein metabolism (7.6%). The antimicrobial properties of this bacterium against several Gram-positive and Gram-negative bacteria, as well as fungal phytopathogens, could be inferred in part with a number of gene inventories encoded in the draft genome. The comparative analysis with closely related bacterial genomes, alignment of the 16S rRNA sequences and prediction of gene inventories for the insecticidal Cry protein biosynthesis placed strain DNG9 under *Bacillus thuringiensis**.* This indicated that strain DNG9 could have several potential utility as an insect biocontrol agent, a fungal phytopathogen control agent, and a source of biopolymers (PHA) and antibacterial compounds. Lastly, the genome sequence of DNG9 may provide another model system to study pathogenicity against insect pests and plant diseases, and for antimicrobial compound mining and phylogenesis among *Bacillus cereus* sensu *lato* group.

## Additional files

Additional file 1: **Figure S1.** Time-course of growth and emulsification index of *B. thuringiensis* DNG9 in LB medium at 27 °C. Time course of growth (*black rhombus*, [OD]) and emulsification index E24 (*grey triangle*, [%]) of *B. thuringiensis* DNG9 during shake flask cultivations in LB medium at 27 °C. The experiments were performed in triplicate and data presented in figure is average of three parallel experiments. Error bars are shown for standard deviation (*P* ≤ 0.05). (DOCX 16 kb)

Additional file 2: **Table S1.** Average nucleotide identity (ANI) and digital DNA:DNA Hybridization (dDDH) between the genome of DNG9 and those of other Bacillales. (XLS 28 kb)

Additional file 3: **Table S2.** Gene inventory of 5 genomic islands in *Bacillus thuringiensis* DNG9 AND seven closely related *Bacillus* sp. (XLS 498 kb)

## Abbreviations

AF: Alignment fraction
antiSMASH: Antibiotics & Secondary Metabolite Analysis SHell
ATCC: American type culture collection
BASys: Bacterial annotation system
Bt corn: *Bacillus thuringiensis* corn
COG: Clusters of orthologous
CRISPR: Clustered Regularly Interspaced Short Palindromic Repeats
DDBJ: DNA Data Bank of Japan
dDDH: Digital DNA:DNA Hybridization
DOE: Department of Energy (United States of America)
ENA: European nucleotide srchive
gANI: Genome-wide Average Nucleotide Identity
GGDC: Genome-to-Genome Distance Calculator
JGI-IMG/M: Joint Genome Institute-Integrated Microbial Genomes and Microbiomes
Mbp: Mega base pair
MeDuSa: Multi-Draft based Scaffolder
MIGS: Minimum Information about a Genome Sequence
MiSI: Microbial Species Identifier
MUCL: Mycotheque de l’Universite Catholique de Louvain
PGAP: Prokaryotic Genome Annotation Pipeline
PHA: Polyhydroxyalkanoate
RAST: Rapid Annotation using Subsystem Technology

## References

[1] Palma L, Muñoz D, Berry C, Murillo J, Caballero P (2014). *Bacillus thuringiensis* toxins: an overview of their biocidal activity. Toxins.

[2] Jouzani GS, Valijanian E, Sharafi R (2017). *Bacillus thuringiensis*: a successful insecticide with new environmental features and tidings. App Microbiol Biotechnol.

[3] Hellmich RL, Hellmich KA (2012). Use and impact of Bt maize. Nat Educ Knowl.

[4] Mendelsohn M, Kough J, Vaituzis Z, Matthews K (2003). Are Bt crops safe?. Nat Biotechnol.

[5] Bravo A, Likitvivatanavong S, Gill SS, Soberón M (2011). *Bacillus thuringiensis*: a story of a successful bioinsecticide. Insect Biochem Mol Biol.

[6] Pardo-Lopez L, Soberon M, Bravo A (2012). *Bacillus thuringiensis* insecticidal three-domain cry toxins: mode of action, insect resistance and consequences for crop protection. FEMS Microbiol Rev.

[7] Djenane Z, Nateche F, Amziane M, Gomis-Cebolla J, El-Aichar F, Khorf H (2017). Assessment of the antimicrobial activity and the entomocidal potential of *Bacillus thuringiensis* isolates from Algeria. Toxins.

[8] Deepak R, Jayapradha R (2015). Lipopeptide biosurfactant from *Bacillus thuringiensis* pak2310: a potential antagonist against *Fusarium oxysporum*. J Mycol Med.

[9] Stein T (2005). *Bacillus subtilis* antibiotics: structures, syntheses and specific functions. Mol Microbiol.

[10] Cochrane SA, Vederas JC (2016). Lipopeptides from *Bacillus* and *Paenibacillus* spp.: a gold mine of antibiotic candidates. Med Res Rev.

[11] Daas MS, Rosana AR, Acedo JZ, Nateche F, Kebbouche-Gana S, Vederas JC (2017). Draft genome sequences of *Bacillus cereus* E41 and *Bacillus anthracis* F34 isolated from Algerian salt lakes. Genome Announce.

[12] Daas MS, Acedo JZ, Rosana AR, Orata FD, Reiz B, Zheng J (2017). *Bacillus amyloliquefaciens* ssp. *plantarum* F11 isolated from Algerian salty lake as a source of biosurfactants and bioactive lipopeptides. FEMS Microbiol Lett.

[13] Sumi CD, Yang BW, Yeo IC, Hahm YT (2014). Antimicrobial peptides of the genus *Bacillus*: a new era for antibiotics. Can J Microbiol.

[14] Baumann L, Okamoto K, Unterman BM, Lynch MJ, Baumann P (1984). Phenotypic characterization of *Bacillus thuringiensis* and *Bacillus cereus*. J Invertebr Pathol.

[15] Barjac H, Frachon E (1990). Classification of *Bacillus thuringiensis* strains. BioControl.

[16] Gibbons NE, Murray RG (1978). Proposals concerning the higher taxa of bacteria. Int J Syst Evol Microbiol.

[17] West AW, Burges H, Dixon TJ, Wyborn CH (1985). Survival of *Bacillus thuringiensis* and *Bacillus cereus* spore inocula in soil: effects of pH, moisture, nutrient availability and indigenous microorganisms. Soil Biol Biochem.

[18] Ye W, Zhu L, Liu Y, Crickmore N, Peng D, Ruan L, Sun M (2012). Mining new crystal protein genes from *Bacillus thuringiensis* on the basis of mixed plasmid-enriched genome sequencing and a computational pipeline. Appl Environ Microbiol.

[19] Helgason E, Økstad OA, Caugant DA, Johansen HA, Fouet A, Mock M (2000). *Bacillus anthracis, Bacillus cereus*, and *Bacillus thuringiensis*—one species on the basis of genetic evidence. Appl Environ Microbiol.

[20] Nakamura LK, Roberts MS, Cohan FM (1999). Relationship of *Bacillus subtilis* clades associated with strains 168 and W23: a proposal for *Bacillus subtilis* subsp. *subtilis* subsp. nov. and *Bacillus subtilis* subsp. *spizizenii* subsp. nov. Int J Syst Bacteriol.

[21] Field D, Garrity G, Gray T, Morrison N, Selengut J, Sterk P (2008). The minimum information about a genome sequence (MIGS) specification. Nat Biotechnol.

[22] Rosana AR, Chamot D, Owttrim GW (2012). Autoregulation of RNA helicase expression in response to temperature stress in *Synechocystis* sp. PCC 6803. PLoS One.

[23] Tatusova T, DiCuccio M, Badretdin A, Chetvernin V, Nawrocki EP, Zaslavsky L (2016). NCBI prokaryotic genome annotation pipeline. Nucleic Acids Res.

[24] Markowitz VM, Chen IM, Palaniappan K, Chu K, Szeto E, Pillay M (2013). IMG 4 version of the integrated microbial genomes comparative analysis system. Nucleic Acids Res.

[25] Hyatt D, Chen GL, LoCascio PF, Land ML, Larimer FW, Hauser LJ (2010). Prodigal: prokaryotic gene recognition and translation initiation site identification. BMC Bioinformatics.

[26] Aziz RK, Bartels D, Best AA, DeJongh M, Disz T, Edwards RA (2008). The RAST server: rapid annotations using subsystems technology. BMC Genomics.

[27] Van Domselaar GH, Stothard P, Shrivastava S, Cruz JA, Guo A, Dong X (2005). BASys: a web server for automated bacterial genome annotation. Nucleic Acids Res.

[28] Grissa I, Vergnaud G, Pourcel C (2007). CRISPRFinder: a web tool to identify clustered regularly interspaced short palindromic repeats. Nucleic Acids Res.

[29] Galardini M, Biondi EG, Bazzicalupo M, Mengoni A (2011). CONTIGuator: a bacterial genomes finishing tool for structural insights on draft genomes. Source Code Biol Med.

[30] Bosi E, Donati B, Galardini M, Brunetti S, Sagot MF, Lió P (2015). MeDuSa: a multi-draft based scaffolder. Bioinformatics.

[31] Grant JR, Stothard P (2008). The CGView server: a comparative genomics tool for circular genomes. Nucleic Acids Res.

[32] Varghese NJ, Mukherjee S, Ivanova N, Konstantinidis KT, Mavrommatis K, Kyrpides NC (2015). Microbial species delineation using whole genome sequences. Nucleic Acids Res.

[33] Meier-Kolthoff JP, Auch AF, Klenk HP, Göker M (2013). Genome sequence-based species delimitation with confidence intervals and improved distance functions. BMC Bioinformatics..

[34] He J, Wang J, Yin W, Shao X, Zheng H, Li M (2011). Complete genome sequence of *Bacillus thuringiensis* subsp. *chinensis* strain CT-43. J Bacteriol.

[35] Murawska E, Fiedoruk K, Bideshi DK, Swiecicka I (2013). Complete genome sequence of *Bacillus thuringiensis* subsp. *thuringiensis* strain IS5056, an isolate highly toxic to *Trichoplusia*. Genome Announce.

[36] Wang P, Zhang C, Guo M, Guo S, Zhu Y, Zheng J (2014). Complete genome sequence of *Bacillus thuringiensis* YBT-1518, a typical strain with high toxicity to nematodes. J Biotechnol.

[37] Schleifer KH, Kraus J, Dvorak C, Kilpper-Bälz R, Collins MD, Fischer W (1985). Transfer of *Streptococcus lactis* and related streptococci to the genus *Lactococcus* gen. Nov. Syst Appl Microbiol.

[38] Schink B, Pfenning N. In Validation List no. 20. Validation of the publication of new names and new combinations previously effectively published outside the IJSB. Int J Syst Bacteriol. 1986;36:354–56.

[39] Collins MD, Farrow JAE, Phillips BA, Ferusu S, Jones D (1987). Classification of *Lactobacillus divergens, Lactobacillus piscicola*, and some catalase-negative asporogenous, rod-shaped bacteria from poultry in a new genus, *Carnobacterium*. Int J Syst Bacteriol.

[40] Le Minor L, Popoff MY (1987). Request for an opinion. Designation of *Salmonella enterica* sp. nov., nom. Rev., as the type and only species of the genus *Salmonella*. Int J Syst Bacteriol.

[41] Cohn F (1872). Untersuchungen über Bakterien. Beiträge zur Biologie der Pflanzen.

[42] Skerman VBD, McGowan V, Sneath PHA (1980). Approved lists of bacterial names. Int J Syst Bacteriol.

[43] Castellani A, Chalmers AJ (1918). Genus *Escherichia* Castellani and Chalmers. Manual Trop Med.

[44] Migula W (1900). *Pseudomonas aeruginosa* (Schröter) Mig. System der Bakterien.

[45] Schleifer KH, Kilpper-Bälz R. Transfer of *Streptococcus faecalis* and *Streptococcus faecium* to the genus *Enterococcus* nom. Rev. as *Enterococcus faecalis* comb. nov. and *Enterococcus faecium* comb. nov. Int J Syst Bacteriol. 1984;34:31–34.

[46] Silo-Suh LA, Stabb EV, Raffel SJ, Handelsman J (1998). Target range of zwittermicin a, an aminopolyol antibiotic from *Bacillus cereus*. Curr Microbiol.

[47] Emmert EA, Klimowicz AK, Thomas MG, Handelsman J (2004). Genetics of zwittermicin a production by *Bacillus cereus*. Appl Environ Microbiol.

[48] Koppisch AT, Dhungana S, Hill KK, Boukhalfa H, Heine HS, Colip LA (2008). Petrobactin is produced by both pathogenic and non-pathogenic isolates of the *Bacillus cereus* group of bacteria. Biometals.

[49] Wilson MK, Abergel RJ, Arceneaux JE, Raymond KN, Byers BR (2010). Temporal production of the two *Bacillus anthracis* siderophores, petrobactin and bacillibactin. Biometals.

[50] Frankland GC, Frankland PF (1887). Studies on some new microorganisms obtained from air. Philos Trans R Soc Lond Ser B Biol Sci.

[51] Bertelli C, Laird MR, Williams KP, Lau BY, Hoad G, Simon Fraser University Research Computing Group (2017). IslandViewer 4: expanded prediction of genomic islands for larger-scale datasets. Nucleic Acids Res.

[52] Arndt D, Grant JR, Marcu A, Sajed T, Pon A, Liang Y, Wishart DS (2016). PHASTER: a better, faster version of the PHAST phage search tool. Nucleic Acids Res.

[53] Woese CR, Kandler O, Wheelis ML (1990). Towards a natural system of organisms: proposal for the domains archaea, bacteria, and Eucarya. Proc Natl Acad Sci U S A.

[54] Validation EJ, No L (2010). 132. List of new names and new combinations previously effectively, but not validly, published. Int J Syst Evol Microbiol.

[55] Ludwig W, Schleifer KH, Whitman WB, De Vos P, Garrity G, Jones D, Krieg NR, Ludwig W (2009). Class I. *Bacilliclass* nov. Bergey’s Manual of Systematic Bacteriology.

[56] Prévot AR, Hauderoy P, Ehringer G, Guillot G, Magrou J, Prévot AR, Rosset D, Urbain A (1953). Dictionnaire des Bactéries Pathogènes.

[57] Fischer A (1895). Untersuchungen über bakterien. Jahrbücher für Wissenschaftliche Botanik.

[58] Berliner E (1915). Über die Schlaffsucht der Mehlmottenraupe (Ephestia kühniella Zell.) und ihren Erreger Bacillus thuringiensis n. sp. J Appl Entomol.

[59] Vilas-Boas GT, Peruca AP, Arantes OM (2007). Biology and taxonomy of *Bacillus cereus, Bacillus anthracis*, and *Bacillus thuringiensis*. Can J Microbiol.

[60] Schnepf E, Crickmore NV, Van Rie J, Lereclus D, Baum J, Feitelson J (1998). Bacillus thuringiensis and its pesticidal crystal proteins. Microbiol Molec Biol Rev.

[61] Ashburner M, Ball CA, Blake JA, Botstein D, Butler H, Cherry JM (2000). Gene ontology: tool for the unification of biology. Nat Genet.

