# Supplementary material for: Insights into the draft genome sequence of bioactives-producing Bacillus thuringiensis DNG9 isolated from Algerian soil-oil slough
Source: Stand Genomic Sci. 2018 Oct 11;13:25. doi: 10.1186/s40793-018-0331-1 (PMC6186030; doi:10.1186/s40793-018-0331-1)
Supplement: Supplementary file 1 — Figure S1. Time-course of growth and emulsification index of B. thuringiensis DNG9 in LB medium at 27 °C. Time course of growth (black rhombus, [OD]) and emulsification index E24 (grey triangle, [%]) of B. thuringiensis DNG9 during shake flask cultivations in LB medium at 27 °C. The experiments were performed in triplicate and data presented in figure is average of three parallel experiments. Error bars are shown for standard deviation (P ≤ 0.05). (DOCX 16 kb) [file 40793_2018_331_MOESM1_ESM.docx]

**Additional File 1 :**

**Fig. S1 Time-course of growth and emulsification index of *B. thuringiensis* DNG9 in LB medium at 27 °C.** Time course of growth (*black rhombus*, [OD]) and emulsification index E24 (*grey triangle*, [%]) of *B. thuringiensis* DNG9 during shake flask cultivations in LB medium at 27 °C. The experiments were performed in triplicate and data presented in figure is average of three parallel experiments. Error bars are shown for standard deviation (P ≤ 0.05).
